# Book Review: Smart Pharmaceutical Nanocarriers

**DOI:** 10.3389/fchem.2017.00112

**Published:** 2017-12-04

**Authors:** Sushilkumar A. Jadhav

**Affiliations:** ^1^Department of Chemistry, University of Torino, Torino, Italy; ^2^NIS Research Centre, University of Torino, Torino, Italy

**Keywords:** nanocarriers, stimuli-responsive nanocarriers, smart nanocarriers, hybrid nanocarreirs, target drug delivery

Nano-sized drug carriers (nanocarriers) have revolutionized the diagnosis and drug delivery related research. Recently, various new advanced material based nanocarriers are synthesized and tested for targeted drug delivery applications. “Smart nanocarriers” are the advanced nanocarriers which respond to different types of stimuli (Ganta et al., 2008; Mura et al., 2013). This area of research has received increased attention due to the proven advantages of the smart nanocarriers over simple nanocarriers. Several new approaches of synthesis and testing of smart nanocarriers for durg delivery are reported recently (Brunella et al., 2016; Ugazio et al., 2016; Jadhav et al., 2017a,b). Smart nanocarriers help in active targeting and deliver the loaded drugs (cargo) at specific site and at required rate by virtue of stimuli-responsive mechanisms. The cargo release can be stimulated or controlled by adjusting the microenvironment conditions around the nanocarrier. The “smart pharmaceutical nanocarriers” book contains detailed information about various stimuli-responsive nanocarriers. The chapters in the book are classified according to the type of external or internal stimuli used to trigger the release of drugs from the smart nanocarriers. The characteristic feature of the book is that all the chapters are written in detail and latest literature sources of the information are cited. This feature makes the book useful source to find collective information about specific stimuli- responsive mechanisms for designing the smart nanocarriers. Numerous examples of nanopreparations for combination therapy and attempts to improve their performance are discussed. Present state of the art and some future challenges to be answered are also discussed. This information is important to decide the direction of future research on smart nanocarriers.

The first chapter is written by the editor and his co-workers which covers the detailed information about various stimuli-responsive nanocarriers used for the delivery of different classes of drugs. A good collection of recent literature regarding various stimuli-responsive nanocarriers is provided. The chapters 2 to 6 are dedicated to pH sensitive, matrix metalloproteinase-sensitive, redox sensitive, temperature sensitive, and ultrasound sensitive nanocarreirs, respectively. The chapter 7 deals with plasmonic nanobubble (PNB)-controlled on demand drug delivery. A comparative evaluation of PNBs with nanoparticles (NPs)-based drug delivery systems (DDSs) and high cellular specificity of PNBs is presented with data from various works. Chapters 8 to 11 are dedicated to light, magnetic field, smart lipid and smart polymer based smart pharmaceutical nanocarriers respectively. The chapter 12 provides collective information about inorganic nanoparticle based smart DDSs. In such DDSs inorganic nanoparticles are used for the designing of nanocarriers which are then functionalized with stimuli responsive mechanisms. Most frequently used inorganic materials such as iron oxide, gold, silver, silica, and porous silica nanoparticles are covered in the discussion. The specific requirements of physicochemical properties such as diameter, surface charge, and reactivity for conjugation of them with molecules for targeted delivery are also discussed. The chapter 13 covers the discussion about smart nanopreparation for diagnosis of cancer and drug delivery for cancer. Smart nanopreparations based on nanoparticles, cell-specific targeting strategies for cancer, and internalization of the particles is discussed. The focus of discussion is on gold and iron oxide nanoparticles based smart diagnostic and DDSs. The chapter 14 deals with smart nanopreparations for oral drug delivery. Advanced nanosystems such as nanocrystals, polymeric nanocarriers and inorganic hybrid systems for oral drug delivery are discussed in detail. The last chapter (15) is dedicated to “smart theranostic nanosystems,” a form of diagnostic testing employed for selecting targeted therapy.

Some other excellent books on similar topic are published recently. They also cover interesting discussion on organic smart materials for drug delivery (Grumezescu, 2018) or separate discussion on internal and external stimuli response of the smart nanocarriers (Karimi et al., 2015a,b). The present book instead provides the collective information on smart nanocarriers based on various materials and discusses both types of stimuli. Therefore it is an excellent book for researchers working on the development of smart nanocarriers based on various materials. It is also useful for academics for designing of the courses for research (M.Phil. and Ph.D. level) students. Periodic editions of the book in future reporting the latest developments in the field of smart pharmaceutical nanocarriers would benefit the scientific community working in this area.

## Author contributions

The author confirms being the sole contributor of this work and approved it for publication.

### Conflict of interest statement

The author declares that the research was conducted in the absence of any commercial or financial relationships that could be construed as a potential conflict of interest.
